# Decreasing corn particle size increases energy and nitrogen digestibility in gestating sows

**DOI:** 10.1093/tas/txag010

**Published:** 2026-01-30

**Authors:** Gage E Nichols, Caitlin E Evans, Chad B Paulk

**Affiliations:** Department of Grain Science and Industry, College of Agriculture, Kansas State University, Manhattan, KS 66506, United States; Department of Grain Science and Industry, College of Agriculture, Kansas State University, Manhattan, KS 66506, United States; Department of Grain Science and Industry, College of Agriculture, Kansas State University, Manhattan, KS 66506, United States

**Keywords:** gestating sows, metabolizable energy, particle size

## Abstract

Results of previous research has demonstrated that reducing the particle size of corn improved metabolizable energy (ME) utilization in many phases of swine production. One phase that has limited research thus far is gestation sows. The objective of this experiment was to determine the effects of corn particle size on the digestibility of protein (CP), and digestible energy (DE), ME, and nitrogen adjusted metabolizable energy (AMEn) in gestating sow diets. A total of 27 sows during the second phase of gestation (day 40 to 74) were fed a common diet with one of 3 target geometric mean diameter (dgw) of corn ground to either 400, 800, or 1200 µm. Corn was ground using a 3 high roller mill (RMS model 924). Titanium dioxide (0.25%) was included in the diet as an indigestible marker for index digestibility calculations. Sows were fed experimental diets for 7 days to allow for diet adaptation before a 2-day collection period. Apparent total tract digestibility (ATTD) of crude protein (CP), and DE, ME, and AMEn increased (linear, *P <*0.001) as corn particle size was reduced from 1200 to 400 µm. The ME and AMEn (88.5% DM) of the diet increased by 178 and 172 kcal/kg, respectively, which resulted in a 22.3 and 21.5 kcal/kg, respectively, improvement for every 100 μm decrease in particle size.

## Teaser

Reducing the particle size of corn from 1200 to 400 µm increased the ME and apparent total tract digestibility of crude protein.

## Introduction

Feed costs represent approximately 70% of the cost of pork production (Patience et al. 2015). Therefore, swine producers strive to reduce the cost of feed by improving performance and nutrient efficiency. One way to achieve this goal is to improve nutrient digestibility through reducing corn particle size. Particle size reduction of grains continues to be used in the swine industry to increase nutrient utilization regardless of production phase (Lancheros et al. 2020). Both roller mills and hammermills are utilized to grind grains. The type of mill used is commonly chosen by capacity, target particle size, and energy efficiency (Hancock and Behnke 2001). Grinding to a reduced particle size can be achieved using either a hammermill or a roller mill. Hammermills reduce particle size through high-impact forces as rotating hammers fracture grains until the particles are small enough to pass through a screen. In contrast, roller mills reduce particle size by applying compressive and shear forces between rotating rolls. Roller mills produce a more uniform particle size compared with hammermills because of impact grinding creating fine particles. Variation in particle size uniformity can result in challenges with poorer flowability (Groesbeck et al. 2006).

Reducing the particle size of corn is a common practice because of its subsequent improvements on pig growth performance (Lancheros et al. 2020). Healy et al. (1994) demonstrated that reducing particle size from 900 to 300 μm during the first 14 days post weaning resulted in an improvement in Gain: Feed (G: F) and average daily gain (ADG). Improvements in G: F have also been demonstrated in finishing pigs as particle size was reduced from 1000 to 400 μm (Wondra et al. 1995b). These improvements in growth performance can be attributed to an increase in surface area to volume ratio of particles in the corn as ­particle size ­decreases. Therefore, this increase in surface area allows for greater enzyme activity which can lead to improvements in utilization (Huang et al. 2015). Results of previous research has demonstrated that decreasing corn particle size (d_gw_) from 1200 to 400 μm improves gross energy (GE), digestible energy (DE), metabolizable energy (ME), and nitrogen (N) digestibility in lactating sows (Wondra et al. 1995a). Healy et al. (1994) observed a 2% increase in apparent total tract digestibility (ATTD) of GE digestibility in nursery pigs. Similar improvements have been reported in finishing pigs with ATTD GE improving by 3%, or 7% as particle size was reduced from 865 to 339 μm or 1000 to 400 μm, respectively (Wondra et al. 1995b; Rojas and Stein 2015).

The majority of particle size research has been conducted using nursery and grow finish pigs, with limited research with lactating sows, and limited research has been conducted to determine effects of corn particle size on nutrient digestibility in gestating sows. Therefore, the objective of this experiment was to determine the effects of corn particle size on the digestibility of dry matter (DM), crude protein (CP), and DE, ME, and nitrogen adjusted metabolizable energy (AMEn) in gestating sow diets. Therefore it was hypothesized that decreasing corn particle size would increase digestibility of DM, CP, DE, ME and AMEn in gestating sow diets.

## Materials and methods

Kansas State University Institutional Animal Care and Use Committee approved all protocols used in this experiment. This experiment was conducted at the Kansas State University Swine Teaching and Research Center in Manhattan, KS.

### Animal housing, diet, and feeding

A total of 27 gestating sows (Line 241; DNA, Columbus, NE) of varying parity’s (15 parity 1, 9 parity 2, and 3 parity 3 sows) were utilized, during the second phase of their gestation (day 40–74). Sows were individually housed in an environmentally controlled room with mechanical ventilation. Sows had ad libitum access to water via a nipple waterer. Sows were fed 2, 2.5, or 3 kg once daily at 0700 h based on a body condition of 2, 3, or 4 respectively. A corn and soybean meal-based diet was formulated based on the NRC 2012 recommended requirement (Table 1). Diets consisted of corn ground to either 400, 800, or 1200 μm using a 3 high roller mill (Model 924, RMS Roller Grinder, Harrisburg, SD). Sows were split into 3 groups based upon breeding date and allotted to dietary treatment within group and balanced by parity and back fat thickness. Average feed intakes for sows were 2.3 kg for 400 μm treatment, 2.4 kg for the 800 μm treatment and 2.4 kg for the 1200 μm treatment. Titanium dioxide (0.25%) was added to the diets as an indigestible marker. Sows were fed for 7 days to allow for adaptation to the treatment diets followed by a two-day collection period of fecal and urine samples.

**Table 1:** Diet composition, as-fed basis.

Ingredient, %	
** Corn**	78.15
** Soybean meal, 46.5%**	17.27
** Soy oil**	0.50
** Monocalcium phosphate 21%**	1.30
** Limestone**	1.30
** Salt**	0.50
** Trace mineral premix** ^a^	0.15
** Vitamin premix** ^b^	0.25
** Sow add pack** ^c^	0.25
** Phytase** ^d^	0.08
** Titanium dioxide**	0.25
**Total**	100
**Calculated analysis** ^e^ **, as-fed basis**	
** Metabolizable energy, kcal/kg**	3265
** Net energy, kcal/kg**	2486
** Crude protein, %**	14.7
** Calcium, %**	0.91
** Phosphorus, %**	0.61
** Calcium: phosphorus**	1.50
**Analyzed nutrients, as-fed basis**	
** Dry matter, %**	89.40
** Gross energy, kcal/kg**	3887
** Crude protein, %**	14.74

a Provided per kg of premix: 3 g Cu from copper sulfate; 160 mg Ca from calcium iodate; 31 mg Fe from ferrous sulfate; 3 g Mn from manganese sulfate; 120 mg Se from sodium selenite; and 31 g Zn from zinc sulfate.

b Provided per kg of premix: 1,543,220 IU vitamin A from vitamin A acetate; 440,920 IU vitamin D from vitamin D3; 8047 IU vitamin E from dl-α-tocopherol acetate; 882 mg menadione from menadione nicotinamide bisulfite; 8 mg B12 from cyanocobalamin; 14,991 mg niacin from niacinamide; 6614 pantothenic acid from d-calcium pantothenate; 1984 mg riboflavin from crystalline riboflavin.

c Provided per kg of premix: 0.077 g chromium, 1,653,750 IU vitamin A from vitamin A acetate; 8820 vitamin E from dl-α-tocopherol acetate; 88.2 mg biotin, 882 mg Folic acid, 367 mg Pyridoxine, 220,500 mg Choline, 19,845 mg Carnitine.

d Ronozyme HiPhos (GT) 2700 (DSM Nutritional Products, Parsippany, NJ) provided 1,102,300 phytase units (FTU)/kg of product with a release of 0.10% available P.

e Calculated nutrient values are based on NRC 2012 values.

### Sample collection

At the start of the collection period each sow was fitted with a Foley catheter (Bard Bardia 2-way, 30 mL balloon, 18 French; Bard Medical Canada Inc., Oakville, ON, Canada) with methods adapted from Holen et al. (2020). While sows were standing, the vulva region was cleaned with antiseptic solution (Betadine), and ­isopropyl alcohol. Technicians washed their hands before working with each sow with antiseptic soap and then wore sterile surgical gloves. Lubricant was placed onto the catheter and the hand of the technician before insertions. The lubricant was laced with lidocaine to prevent urethral spasms during placement. A total of 5 min was allotted for each placement to help prevent infection. Should a sow not be fitted with a catheter within the allotted time, she was removed from test. There were 9 sows placed on the 800 and 1200 μm treatments and 8 sows placed on the 400 μm treatment as one sow was not able to be catheterized within the allotted placement time. The tip of the catheter was guided by the technician’s finger along the floor of the vagina until it entered the urethra. Once the catheter was fully inserted the 30 mL balloon was inflated with 30 mL of saline solution to retain the catheter in the bladder. Polyvinyl tubing was connected to the catheters that dispensed the urine into a collection vessel. Sulfuric acid (20 mL) was added to each bucket to keep the pH below 3 to limit bacterial growth and maintain nitrogen levels. Urine was weighed, and collection vessels were emptied, and subsamples were collected to obtain 20% aliquots. Aliquots were stored separately at −20°C and subsamples were pooled within sow at the end of the experiment. At the end of the 48-hour collection period the 30 mL balloon was deflated and the catheter was removed. During collection periods sow behavior and vaginal discharge was evaluated twice daily. Sow temperatures were also collected twice daily during the collection period, and for 5 days after the collection period to monitor for any signs of urinary tract infection.

Fecal grab samples were collected throughout the collection period for each sow, bagged and stored separately at −20°C. Prior to fecal sample collection, gestation crates were cleaned using high pressure water. Fecal samples were collected twice daily. Fecal grab samples were collected as the sow was defecating or fresh samples from the floor directly behind the sow were collected. At the end of the experiment, fecal samples were pooled within sow. All samples were stored −20°C until the end of the collection period. At the end of the experiment samples were pooled within sow and subsamples were collected for analysis.

### Sample analysis

To determine the d_gw_ and geometric standard deviation (S_gw_) of both the corn used in the diets and the diets, the rotap 13 sieve method utilizing 0.5 g sieving agent with a 10-minute run time was used (ANSI/ASAE method S319.2,1996).

To determine DM of diets, aluminum pans were weighed, and a ground sample was placed in the drying oven at 105°C for 24 h. Samples were weighed back for moisture calculation (100—dried sample weight/initial sample weight × 100; AOAC Method 934.01). Fecal DM was determined in a two-step process. Samples were weighed and dried in a forced-air oven at 55°C for 48 h. Samples were then weighed and moisture loss was recorded. Samples were ground to be used for nutrient analysis and final DM analysis. To determine final dry matter of the fecal samples, aluminum pans were weighed, and a ground sample was weighed and placed in a forced air oven at 105°C for 24 h in duplicate. Dried sample weights were recorded, and moisture levels were added from both drying steps to determine overall DM.

Fecal samples, urine, and diets were analyzed for N via combustion using a Leco (Model FP928, St Joseph, MI, Method 990.03; AOAC, 2007) nitrogen analyzer. Feed and fecal gross energy was determined via bomb calorimetry by ATC Scientific (North Little Rock, AR) using a Parr model 6100 (Parr Instrument Company, Moline, IL) bomb calorimeter. Urine samples were analyzed utilizing a Parr model 6200 (Parr Instrument Company, Moline, IL) bomb calorimeter using methods adapted from Jones (2015). To analyze urine for energy, cellulose was ground and dried at 105°C for 24 h and stored in a sealed bag until use. Then, 1 g of cellulose was placed into a bomb cup and 4 mL of urine was pipetted over the cellulose. The cup with cellulose and urine was dried for 12 h in a forced air oven at 55°C. Cups were removed from the oven and placed in a desiccator until they were bombed. Fecal and feed samples were analyzed for titanium dioxide via the methods described by Leone (1973).

#### Calculations and statistical analysis

The DE and ME in each diet were calculated using the following equations:


$$\mathrm{DEdiet}=1-\left(\frac{{\%\;\mathrm{TiO}2}_{\mathrm{diet}}\;}{{\%\;\text{TiO}2}_{\mathrm{feces}}}\right.\;\times \left.\frac{{GE}_{\mathrm{feces}}}{{GE}_{\mathrm{diet}}}\right)$$

$${ME}_{\mathrm{Diet}}={DE}_{\mathrm{Diet}}-{GE}_{\mathrm{Urine}}$$

where GE_Diet_, GE_Feces_, GE_Urine,_ TiO2_Diet_, TiO2_Feces_ represent the concentration of energy (kcal/kg) in the diet, fecal samples, and urine sample and titanium dioxide (%) in the diet and urine samples respectively.

The apparent total tract digestibility (ATTD) of each diet was calculated using the following equation:


$${\mathrm{ATTD}}_{\mathrm{Nutrient}},\%=\;1-(\frac{{\%\;\mathrm{TiO}2}_{\mathrm{diet}}\;}{{\%\;\text{TiO}2}_{\mathrm{feces}}})\;\times (\frac{{\mathrm{Nutrient}}_{\mathrm{feces}}}{{\mathrm{Nutrient}}_{\mathrm{diet}}})\times 100$$

where Nutrient_diet_ and Nutrient_feces_ represent the percent of the nutrient of interest in the diet and feceses, respectively.

Lastly, N retention (Nr) was calculated using the following set of equations:


N intake, g/d=feed intake during collection period, g/d×(feed N content, % ÷ 100)

$$\mathrm{Fecal}\;N,\;g/d=N\;\mathrm{intake},\;g/d\times \left(1—\mathrm{ATT}D_{N}÷\;100\right)$$

Urinary N, g/d=urine weight, g/d×(urine N content, % ÷ 100)

Nr, g/d=N intake, g/d—Fecal N, g/d—Urinary N, g/d

N retention, % of intake=(N retained g/d ÷ N intake, g/d)×100

The ME was corrected for retained nitrogen (AMEn) by using the adjustment factor of 7.45 (Harris et al. 1972) and the following calculation:


$$\mathrm{AMEn}\;\left(\mathrm{kcal}/kg\right)=ME_{\mathrm{Diet}}-\left(7.46\times Nr\right)$$

Where Nr is the nitrogen retention, g/kg DMI (Zhang and Adeola 2017).where N intake, fecal N, and urinary N represent the daily intake, fecal output, and urine output (g/d) of N, respectively.

Data were analyzed using the PROC Glimmix procedure of SAS version 9.4 (SAS Institute, Inc., Cary, NC) utilizing linear and quadratic polynomial contrast with sow as the experimental unit and treatment as the fixed effect. Results were considered significant if *P ≤* 0.05 and marginally significant if *P ≤*0.10.

## Results and discussion

Particle size reduction continues to be used in the swine industry to increase nutrient utilization regardless of production phase (Lancheros et al. 2020). Both roller mills and hammermills are utilized to grind grains. The type of mill used is commonly chosen by capacity, target particle size, and energy efficiency (Hancock and Behnke 2001). For the current experiment, a 3 high roller mill was utilized to achieve the target particle sizes of 400, 800, and 1200 μm (Table 2). The d_gw_, or particle sizes of 403, 823, and 1372 μm, were achieved to correspond to the targeted treatments of 400, 800, and 1200 μm, respectively. The S_gw_ for each treatment were 2.59, 2.80, and 2.83, respectively. The d_gw_ of diets were 448, 733, and 1155 and the S_gw_ were 2.52, 2.80, and 2.98, respectively. As corn is ground to a coarser particle size, it is expected that the standard deviation will increase likely due to larger roll gaps in the larger particle size treatments that allow a wider range of particle sizes to flow through (Wondra et al. 1995c; Rojas and Stein 2015). The target particle size of the diet can vary from that of corn as the particle size of other ingredients often does not match that of corn (Wondra et al. 1995c).

**Table 2:** Geometric mean diameter (d_gw_) and geometric standard deviation (s_gw_) of corn and the final diets.^a^^,^^b^

Target corn particle size	400 µm	800 µm	1200 µm
**Corn particle size**			
** d_gw_**	403	823	1372
** S_gw_**	2.59	2.80	2.83
**Diet particle size**			
** d_gw_**	447	732	1154
** S_gw_**	2.52	2.80	2.98

a Diets were ground on a 3 high RMS roller mill (Model 924, RMS Roller Grinder, Harrisburg, SD).

b Analyses were conducted via the ANSI/ASAE method S319.2 (1995) utilizing 0.5 g of sieving agents and a 10 min run time.

Throughout the experiment, sows remained healthy with no signs of urinary tract infection related to being catheterized. One sow was removed from the experiment due to the catheter placement time exceeding the 5 min limit. All DE, ME, and AMEn calculations were conducted on a DM basis. When determining the ME and AMEn, these values were adjusted to an as is basis using a DM of 88.5%.

There was no evidence of difference in the N intake of pigs fed dietary treatments (Table 3). However, pigs fed diets with increasing corn d_gw_ had increased (linear, *P <*0.006) fecal N. There was no evidence of difference in Urinary N. There was no evidence of difference in N retained N (g/d) in pigs fed diets with varying corn d_gw_. However, there was a tendency (quadratic, *P = *062) for N retention as a percentage of intake to decrease when corn dgw was increased form 800 to 1200 μm. Therefore, the ATTD of CP decreased (linear, *P <*0.001) from 84% to 79% as corn particle size increase from 400 to 1200 μm (Table 3). Wondra et al. (1995a, 1995c) observed similar improvements from 6–8% points in CP digestibility in sows when reducing corn particle size from 1200 to 400 μm. Similarly, in both nursery and grow/finish phases, improvements in CP digestion have also been observed ranging from 3 to 17% depending on reduction in particle size (Healy et al. 1994; Kim et al. 2002; Acosta et al. 2020). The improvement in CP digestion could be a result of particle size providing a greater surface area for protease activity (Oryschak et al. 2002). In contrast to what was seen in total tract digestibility, Rojas and Stein (2015) did not observe an improvement in ileal CP digestibility when reducing corn particle size in growing pigs. However, Owsley et al. (1981) determined that both the ileal and total tract CP digestibility of sorghum improved as sorghum particle size was reduced.

**Table 3:** Effect of ground corn geometric mean diameter (d_gw_) on nitrogen utilization and apparent total track digestibility (ATTD) of crude protein (CP) and gross energy (GE), digestible energy (DE), metabolizable energy (ME) and nitrogen corrected metabolizable energy (AMEn) of gestating sows.^a^

Item	Corn d_gw_				Probability, *P*	
	400 µm	800 µm	1200 µm	SEM	Linear	Quadratic
**N intake, g/d**	55.0	56.3	57.1	3.72	0.676	0.953
**Fecal N, g/d**	8.7	9.9	11.8	0.72	0.006	0.645
**Urinary N, g/d**	13.4	10.3	13.0	2.01	0.877	0.163
**N retained, g/d**	32.9	36.2	32.3	3.80	0.893	0.279
**N retention, % of intake**	59.6	64.2	56.8	2.82	0.476	0.078
**ATTD CP, %**	84.1	82.3	79.1	1.00	0.001	0.553
**ATTD GE, %**	86.6	84.6	82.6	1.01	0.006	0.971
**DE, kcal/kg** ^b^	3332	3249	3184	37.3	0.008	0.836
**ME, kcal/kg** ^b^	3248	3181	3070	40.5	0.004	0.646
**AMEn, kcal/kg** ^b^	3154	3081	2982	36.4	0.004	0.784

a A total of 27 sows during the second phase of gestation (day 40 to 74) were fed a common diet with one of 3 target d_gw_ of corn ground to either 400, 800, or 1200 µm.

b DE, ME, and AMEn values were corrected for 88.5% DM.

The ATTD of GE, DE, ME, and AMEn improved (linear, *P <*0.01) as d_gw_ was decreased from 1200 to 400 μm. The experiment demonstrated a 170 and 178 kcal/kg increase in DE and ME, respectively. Wondra et al. (1995a) demonstrated increases in DE and ME of a 344 and 346 kcal/kg, respectively of corn particle size was reduced from 1200 to 400 μm when fed to lactating sows. When correcting ME for nitrogen (AMEN, 88.5% DM) the dietary energy increased by 172 kcal/kg, which resulted in a 21.5 kcal/kg improvement for every 100 μm in d_gw_ decrease in particle size. Bertol et al. (2017) determined in growing-finishing pigs that as corn particle size was reduced from 982 to 525 μm, AMEn of corn increased by 50 kcal/kg for every 100 μm in d_gw_. This value is greater than that observed in the present experiment. However, Rojas and Stein (2015) determined that the ME value of corn increased by 22.9 kcal/kg for every 100 μm reduction in corn d_gw_ when fed to growing pigs. Improvements in energy digestibility are likely due to the starch portion of the corn having a larger surface area allowing for increased access for enzymes such as α-amylase (Huang et al. 2015; Rojas and Stein 2015).

In conclusion, as corn particle size decreases by 100 μm a 22.3 kcal/kg improvement in dietary ME (88.5% DM) was demonstrated in diets fed to gestating sows. The AMEn (88.5% DM) of the diet increased by 172 kcal/kg, which results in a 21.5 kcal/kg improvement for every 100 μm decrease in particle size. Overall, the reduction of corn particle size from 1200 to 400 μm resulted in a 5.9% improvement in CP digestibility and a 5.5% improvement in the dietary ME.
